# Exploring the Potential of Cannabinoids in the Treatment of Tourette’s Syndrome

**DOI:** 10.1192/j.eurpsy.2024.1295

**Published:** 2024-08-27

**Authors:** F. Cunha, I. Santos, N. Castro, R. Andrade, E. Almeida, J. Abreu, J. Martins, R. Vaz, S. Borges

**Affiliations:** Centro Hospitalar Tondela-Viseu , Viseu, Portugal

## Abstract

**Introduction:**

Tourette’s syndrome (TS) is a disorder characterized by repetitive, involuntary movements, and vocalizations known as tics. While there are existing treatment options, there is a growing need for novel pharmacological approaches to manage the symptoms of TS effectively. This study delves into the emerging field of using cannabinoids as a potential treatment for Tourette’s syndrome.

**Objectives:**

The primary objectives of this review are to examine the current evidence base for the use of cannabinoids in the treatment of Tourette’s syndrome, to assess the biological rationale supporting the use of cannabinoids in managing tic severity, to provide insights into the results of existing clinical trials involving cannabinoids and Tourette’s syndrome, and to draw conclusions regarding the potential efficacy and safety of cannabinoid-based treatments for TS.

**Methods:**

Narrative review of the available scientific literature.

**Results:**

There is a strong biological rationale for how cannabinoids could impact tic severity. The endocannabinoid system plays a crucial role in regulating various physiological processes, including motor control and neurotransmitter release. Activation of cannabinoid receptors in the brain may modulate these processes, potentially reducing tics. While limited, two small randomized, placebo-controlled trials of THC have been conducted in TS patients. These trials suggested potential benefits of cannabis-derived agents in reducing tic frequency and severity. Self-report and examiner rating scales demonstrated significant improvements in tic symptoms. The trials indicated that THC treatment did not result in significant adverse effects in TS patients.

**Conclusions:**

The exploration of cannabinoids as a treatment option for Tourette’s syndrome is promising but requires further investigation. The biological mechanisms through which cannabinoids may affect tic severity in TS are sound, suggesting their potential as a therapeutic option. Existing trials with THC have shown encouraging results, demonstrating a reduction in tics without significant adverse effects. However, the limited number of trials warrants caution in drawing definitive conclusions. Despite the promising findings, the overall efficacy and safety of cannabinoid-based treatments remain largely unknown. Further trials are essential to address dosing, active ingredients, optimal administration, and potential long-term effects. Clinical use should be approached with caution. While early evidence is encouraging, additional rigorous studies are needed to establish the safety and efficacy of cannabinoid-based treatments for this disorder.

**Disclosure of Interest:**

None Declared

